# Predicting high-risk pre-capillary pulmonary hypertension: an echocardiographic multiparameter scoring index

**DOI:** 10.1186/s12872-024-04053-0

**Published:** 2024-07-25

**Authors:** Yanan Zhai, Aili Li, Xincao Tao, Qian Gao, Wanmu Xie, Yu Zhang, Aihong Chen, Chi Wang, Jieping Lei, Shangwei Ding, Yantong Cai, Zhenguo Zhai

**Affiliations:** 1 Department of Cardiology, China-Japan Friendship Hospital, No. 2, East Yinghua Road, Chaoyang District, Beijing, 100029 China; 2 Institute of Respiratory Medicine, Chinese Academy of Medical Sciences, Beijing, China; 3 National Clinical Research Center for Respiratory Diseases, Beijing, China; 4 Department of Pulmonary and Critical Care Medicine, China-Japan Friendship Hospital, Beijing, China; 5 Heart Health Research Center, Beijing, China; 6 Data and Project Management Unit, Institute of Clinical Medical Sciences, China-Japan Friendship Hospital, Beijing, China; 7 Department of Ultrasound, The First Affiliated Hospital of Guangzhou Medical University, Guangzhou, Guangdong Province China

**Keywords:** Echocardiography, Pulmonary hypertension, Right ventricule, Scoring index, risk stratification

## Abstract

**Background:**

The risk stratification of pulmonary arterial hypertension proposed by the European Society of Cardiology /European Respiratory Society guidelines in 2015 and 2022 included two to three echocardiographic indicators. However, the specific value of echocardiography in risk stratification of pre-capillary pulmonary hypertension (pcPH) has not been efficiently demonstrated. Given the complex geometry of the right ventricular (RV) and influencing factors of echocardiographic parameter, there is no single echocardiographic parameter that reliably informs about PH status. We hypothesize that a multi-parameter comprehensive index can more accurately evaluate the severity of the pcPH. The purpose of this study was to develop and validate an echocardiographic risk score model to better assist clinical identifying high risk of pcPH during initial diagnosis and follow-up.

**Methods:**

We studied 197 consecutive patients with pcPH. A multivariable echocardiographic model was constructed to predict the high risk of pcPH in the training set. Points were assigned to significant risk factors in the final model based on β-coefficients. We validated the model internally and externally.

**Results:**

The echocardiographic score was constructed by multivariable logistic regression, which showed that pericardial effusion, right atrial (RA) area, RV outflow tract proximal diameter (RVOT-Prox), the velocity time integral of the right ventricular outflow tract (TVI_RVOT_) and S’ were predictors of high risk of pcPH. The area under curve (AUC) of the training set of the scoring model was 0.882 (95%CI: 0.809–0.956, *p* < 0.0001). External validation was tested in a test dataset of 77 patients. The AUC of the external validation set was 0.852. A 10-point score risk score was generated, with scores ranging from 0 to 10 in the training cohort. The estimate risk of high risk of pcPH ranged from 25.1 to 94.6%.

**Conclusions:**

The echocardiographic risk score using five echocardiographic parameters could be comprehensive and useful to predict the high risk of pcPH for initial assessment and follow-up.

## Introduction

Pre-capillary pulmonary hypertension (pcPH) is a haemodynamic condition of pulmonary hypertension (PH), which is defined as resting mean pulmonary arterial pressure (mPAP) > 20 mmHg, pulmonary artery wedge pressure (PAWP) ≤ 15 mmHg and pulmonary vascular resistant (PVR) > 2 WU according to recent recommendations [1]. pcPH is characterized by the occurrence of pulmonary vascular lesions with increased PVR which includes pulmonary arterial hypertension (PAH), chronic thromboembolic PH (CTEPH) and PH due to other diseases. Clinically, it is very important to judge the severity of pcPH and identify high-risk patients for prognosis and treatment. The risk stratification proposed in the guidelines is for patients with PAH. In the 2015 European Society of Cardiology (ESC)/European Respiratory Society (ERS) guidelines for the diagnosis and treatment of PH, risk assessment for PAH was based on a multiparametric approach using a three-strata model to classify patients at low, intermediate, or high risk of death [2]. Truncated or simplified risk stratification models derived from the ESC/ERS model were validated in real-life conditions for PAH and CTEPH [3–6]. In the 2022 ESC/ERS guidelines, a four-strata risk-assessment tool based on refined cut-off levels for WHO-FC, 6MWD, and NT-proBNP have proposed to be more convenient for follow-up [1]. The three-strata risk assessment in the 2022 ESC/ERS guidelines included three echocardiographic parameters: right atrial (RA) area, TAPSE/sPAP, and pericardial effusion. However the value of echocardiography in risk stratification has not been efficiently demonstrated. Echocardiography is a widely used tool for assessing RV structure and function in PH. Given the complex geometry of the right ventricular (RV) and influencing factors of echocardiographic parameter, there is no single echocardiographic parameter that reliably informs about PH status. Therefore, multiparameter comprehensive echocardiographic assessment is necessary for risk stratification of pcPH. And it may be more valuable to add echocardiographic indicators to the four-strata risk-assessment at follow-up.

Thus, the purpose of this study was to: (i) investigate which echocardiographic indicators are more valuable for identifying high risk pcPH; and (ii) develop and validate an echocardiographic scoring model to better assist clinical risk stratification at initial diagnosis and follow-up.

## Methods

### Study population

**Fig. 1:**
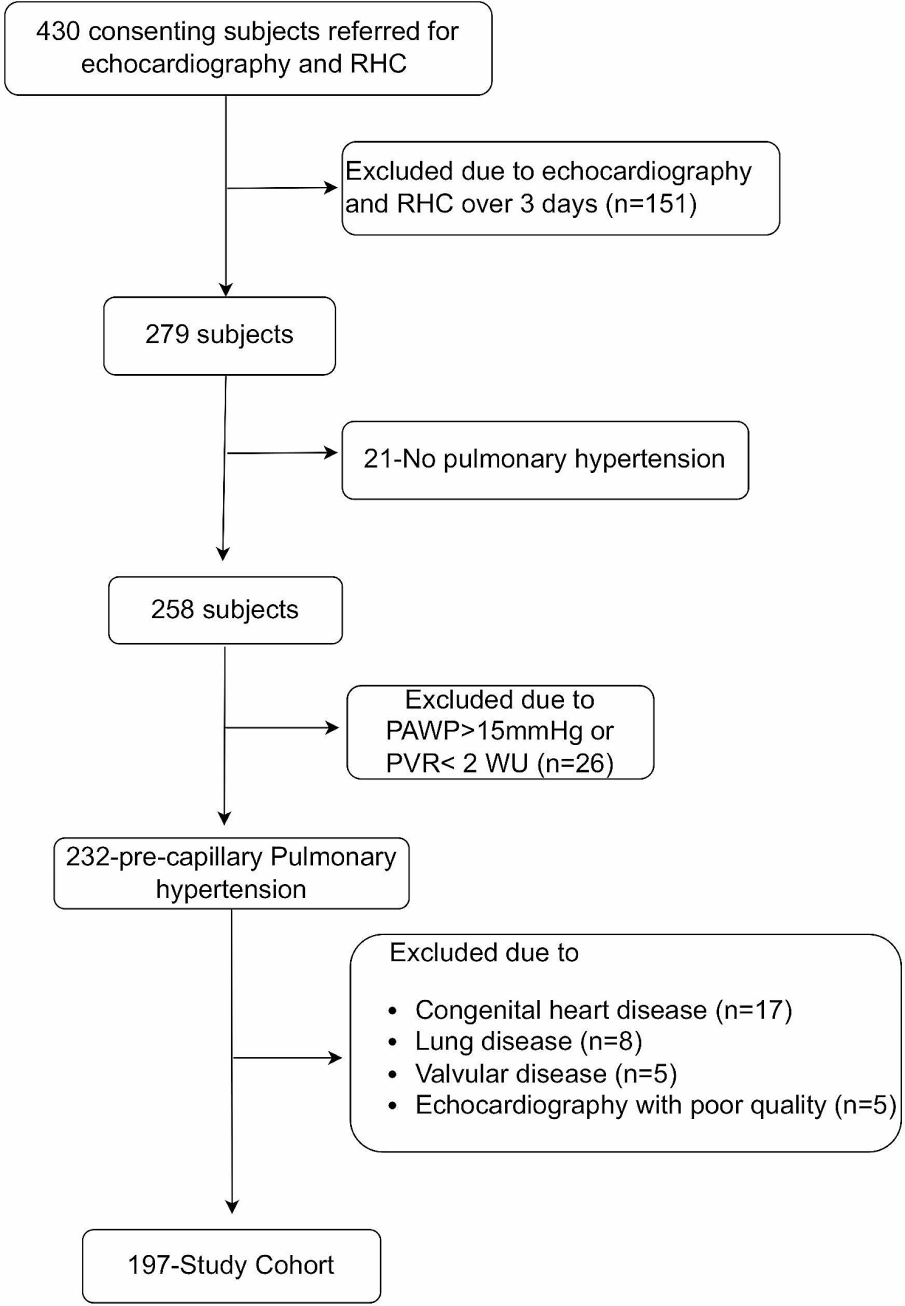
Study flow diagram

A total of 430 consecutive patients referred for transthoracic echocardiography and right heart catheterization (RHC) evaluation of known or suspected PH between June 2016 and December 2021 were included. A subsequent of 169 patients was excluded due to an interval over 3 days between RHC and echocardiography. Of the remaining 244 patients, 223 were diagnosed with PH. pcPH was defined as mPAP > 20 mmHg at rest, pulmonary arterial wedge pressure ≤ 15 mmHg and PVR > 2 Wood units [1]. We excluded patients with one of the followings: patients with lung disease; primary valvular disease; either stenosis of right ventricular outflow tract or pulmonary artery; echocardiography images were of poor quality. In all, 197 pcPH patients were enrolled in the study (Fig. 1). Table 1 described the diagnosis of all the participants according to 2022 ESC/ERS Guidelines [1]. This study was approved by China-Japan Friendship Hospital’s Human Research Ethics Committee.

**Table 1:** Baseline clinical characteristics of the overall analyzed cohort

Baseline characteristics	Development Cohort			
	All subjects (*n* = 197)	Low-intermediate risk (*n* = 160)	High risk (*n* = 37)	*P* value
Demographic characteristics				
Age (yaer)	50(35.5–61.0)	49.5(35.0-61.7)	54.0(42.5–61.0)	0.293
Men, n (%)	80(40.6)	64(40)	16(43.2)	0.717
Heart rate (bpm)	78.7 ± 13.9	77.9 ± 13.9	82.9 ± 13.6	0.066
BSA (m^2^)	1.6(1.5–1.8)	1.6(1.5–1.8)	1.6(1.4–1.7)	0.172
Aetiology of PH, n (%)				
IPAH	37(18.7)	30(18.7)	7(18.9)	
HPAH	2(1.0)	2(1.2)	0(0)	
Portal hypertension	4(2.0)	4(2.5)	0(0)	
PVOD/PCH	16(8.1)	14(8.7)	2(5.4)	
Connective tissue disease	31(15.7)	26(16.2)	5(13.5)	
CTEPH	99(50.2)	78(48.7)	21(56.7)	
Arteritis	7(3.5)	5(3.1)	2(5.4)	
WHO functional class, n (%)				**< 0.001**
I/II	103(52.2)	96(60.0)	7(18.9)	
III/IV	94(47.8)	64(40.0)	30(81.1)	
6MWD (mm)	373 ± 103	380 ± 97.7	177 ± 109.6	**0.001**
NT-proBNP (pg/mL)	608(206–1627)	455.0(163.0-835.2)	2311(1858–3813)	**< 0.001**
PH medical treatment, n (%)				
Phosphodiesterase type-5 inhibitors	18(9.1)	9(5.6)	9(24.3)	**0.001**
Endothelin receptor antagonists	42(21.3)	35(21.8)	7(18.9)	0.692
soluble guanylate cyclase stimulator	13(6.6)	9(5.6)	4(10.8)	0.437
Diuretics	181(91.4)	144(90)	37(100)	0.094

BSA, body surface area; PH, pulmonary hypertension; IPAH, idiopathic pulmonary arterial hypertension; HPAH, heritable pulmonary arterial hypertension; PVOD, pulmonary veno-occlusive disease; PCH, pulmonary capillary haemangiomatosis; CTEPH, chronic thromboembolic pulmonary hypertension; TIA, transient ischemic attacks; 6MWD, 6-minute walking distance; NT-proBNP, N-terminal fragment of pro-B-type natriuretic peptide;

### Risk stratification

We identified high-risk subjects according to a simplified risk stratification in pulmonary artery hypertension (PAH), which was an abbreviated version of the 2015 European Society of Cardiology (ESC)/European Respiratory Society (ERS) risk stratification strategy [3–6] and also supported by 2021 Chinese guidelines for diagnosis and treatment of PH [7]. This simplified method made the risk stratification more clear, simple and convenient for clinical application. Patients were categorized as low, intermediate or high risk. The risk evaluation was based on six parameter: WHO functional class, 6MWD, NT-pro-BNP or BNP plasma levels, cardiac index (CI), mean right atrial pressure (RAP), oxygen saturation of mixed venose blood (SvO_2_). High risk variables included WHO functional class IV; 6MWD < 165 m; BNP > 300ng/L, NT-proBNP > 1400ng/L or RAP > 14mmHg; CI < 2.0 L/min/m^2^ or SvO_2_ < 60%. Patients with at least 2 high risk variables including CI or SvO_2_ were defined as high risk.

### Right heart catheterization

A 7 F Swan-Ganz catheter Philips Allura X-PER FD20 flat-plate angiography system (Baxter Inc) was used to measure systolic, diastolic, and mean pulmonary arterial pressure (PAP), RAP, and mean pulmonary capillary wedge pressure (PCWP). Cardiac output was measured using the Fick method, which calculated CI. The transpulmonary gradient (TPG) was calculated by subtracting the mean PAP from the PCWP. PVR (dyn·s·cm^− 5^) was calculated by dividing the TPG by the cardiac output.

### Standard echocardiography

**Fig. 2:**
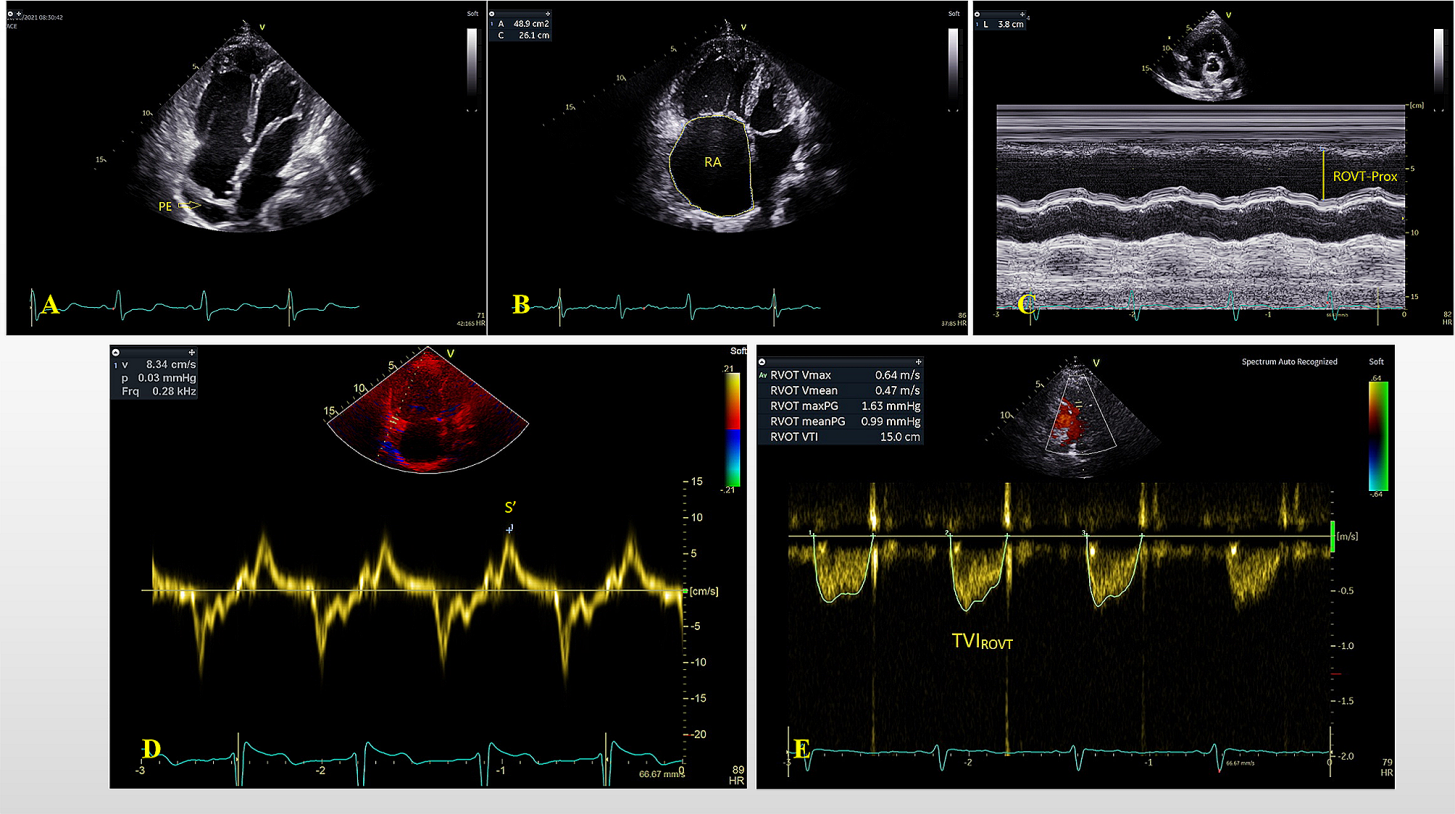
Five echocardiographic parameters to be measured in echocardiographic scoring system. **A.** pericardial effusion (PE) **B.** Right atrial (RA) area **C.** Right ventricular outflow tract proximal diameter (RVOT-Prox) **D.** Systolic annular tissue velocity of the lateral tricuspid annulus (S’) **E.** The velocity time integral of the right ventricular outflow tract (TVI_RVOT_)

Echocariographic examination was performed within 3 days of RHC by two experienced dedicated cardiologists (Aili Li and Yanan Zhai), using the Vivid E95 ultrasound system (General Electric Healthcare, Vingmed, Horten, Norway) equipped with a M5S transducer. Two-dimensional (2D) and Doppler echocardiography were performed according to current guidelines [8]. Analysis of the images was performed offline using the EchoPac software version 201 (General Electric Healthcare, Vingmed, Horten, Norway). Specific related parameters were as follows: LV internal diameter at end-diastole (LVIDd), area of the right atrium (RA), RV basal diameter, right ventricular outflow tract proximal diameter (RVOT-Prox), the velocity time integral of the right ventricular outflow tract (TVI_RVOT_). The severity of TR was graded as non or trace, mild, moderate and severe. The peak tricuspid regurgitation velocity (TRV) and early diastolic pulmonary regurgitation velocity (PREDV) were measured as the highest of the velocities. Noninvasive estimation of RAP was based on the size and collapse index of the IVC. sPAP_echo_ and mPAP_echo_ were calculated by adding the estimated RAP to tricuspid regurgitation pressure gradient (TRPG) and early diastolic pulmonary regurgitation pressure gradient (PREDG) respectively. RV function were evaluated by measuring the tricuspid annular plane systolic excursion (TAPSE), systolic annular tissue velocity of the lateral tricuspid annulus (S’) and RV fractional area change (FAC). (Fig. 2)

### Statistical analysis

Continuous variables were described as the mean ± SD for normally distribution and median(interquartile range)for skewed distribution. The distribution of variables was tested for normality using the Shapiro-Wilks test. Categorical variables were described by frequency and percentage.

Pearson correlation coefficient between the echocardiography parameters was calculated. The variables with *R* > 0.75 were defined as strongly relative parameters. In each pair of strongly relative parameters, the most common one would use for further analysis, which is decided by two experienced cardiologists.

Included parameters were defined as binary variables based on the Cut-off point. Binary variables were used to model the probability of high risk of pcPH by logistic regression. Two steps were used to find independent parameters. Firstly, univariate logistic regression was used. *P* ≤ 0.05 was considered statistically significant. Secondly, a backward selection procedure was used to find independent predictors with stay *p* < 0.2. The receiver-operating characteristic (ROC) method was used to assess the ability of independent variables to predict the high risk of pcPH. The sensitivity and specificity were calculated. The β-coefficients are used to compute the risk score use the method published in 1999 [10].

The calibration plot demonstrates the agreement between observed outcomes and predictions. The bootstrapping method was used to internal verify the model (*n* = 500 replicates). External validation was tested in a test dataset of 77 patients.

All statistical methods were performed using SAS version 9.4 (SAS Institute, NC).

## Results

### Baseline clinical characteristics of the study population

For this analysis, baseline data from a total of 197 patients with fulfilling the inclusion criteria were available. Patients with CTEPH formed the largest subgroup (*n* = 99; 50%), followed by patients with IPAH (*n* = 37; 18%), patients with connective tissue disease (*n* = 31, 15%) and patients with other forms of PH (*n* = 30; 15%). The characteristics of these patients are shown in Table 1.

### Echocardiography and haemodynamic findings of the study population

The echocardiographic and hemodynamic parameters between low-intermediate risk and high risk of pcPH were compared in Table 2. Mean RAP, PVR, CI and SvO_2_ were significantly higher in high risk pcPH than in low-intermediate risk pre-capillary PH (both *p* < 0.001). However, there were no significant differences between groups in systolic PAP and Mean PAP (both *p* > 0.05). The similar results were also found in sPAP_Echo_ and mPAP_Echo_(both *p* > 0.05). In the morphology of the right heart by echocardiography, high risk pcPH had larger RA and RV dimension (RA area, RV diameter, RV area, RV/LV), smaller LV dimension (LVIDd) than low-intermediate risk pcPH (both *p* < 0.001). There were no significant differences between groups in pulmonary artery width and right ventricular wall thickness (both *p* > 0.05). In RV function by echocardiography, TAPSE, S’ and RV FAC were significantly lower in high risk pcPH (*p* < 0.001, *p* = 0.001, *p* = 0.014, respectively). The TR severity and incidence of pericardial effusion in high risk pcPH is higher than that of low-intermediate risk PH (both *p* < 0.001).

**Table 2:** RHC and echocardiography parameters of the overall analyzed cohort

Parameter	Development Cohort			
	All subjects (*n* = 197)	Low-intermediate risk (*n* = 160)	High risk (*n* = 37)	*P* value
Right heart caheterization				
Systolic PAP (mmHg)	78(62.0-93.5)	75(60.2–91.0)	82(72.0-94.5)	0.089
Mean PAP (mmHg)	45.3 ± 14.0	44.6 ± 14.0	48.3 ± 13.8	0.148
Mean RAP (mmHg)	3.2 ± 4.9	2.4 ± 4.0	6.7 ± 6.5	**< 0.001**
PAWP (mmHg)	6.9 ± 4.0	6.8 ± 3.9	7.4 ± 4.4	0.418
PVR (WU)	12.5 ± 7.5	11.1 ± 6.0	18.6 ± 10.0	**< 0.001**
CI (L/min/m^2^)	2.2 ± 0.9	2.5 ± 0.9	1.5 ± 0.3	**< 0.001**
SvO_2_ (%)	68(60.2–74.1)	69(63–76)	57.4(49.3–61.7)	**< 0.001**
Echocardiography				
LVEF (%)	70(66–73)	70(66–73)	72(64–73)	0.267
LA (mm)	33(20.1–36.0)	33(29.2–36.0)	32(28.5–36.0)	0.889
LVIDd (mm)	40.1 ± 5.4	40.7 ± 5.2	37.1 ± 5.2	**< 0.001**
RA minor-axis dimension (mm)	49.3 ± 10.6	47.7 ± 9.1	57.8 ± 11.8	**< 0.001**
RA area (cm^2^)	24.1 ± 9.7	22.2 ± 8.5	31.8 ± 10.9	**< 0.001**
RV basal diameter (mm)	45.9 ± 6.9	44.5 ± 6.3	51.3 ± 6.9	**< 0.001**
RVEDA (cm^2^)	25.8 ± 7.4	24.3 ± 6.6	31.3 ± 7.9	**< 0.001**
RVESA (cm^2^)	17.8(13.4–21.8)	16.3(12.4–20.3)	22.6(18.4–27.2)	**< 0.001**
RV/LV basal diameter ratio	1.2(1.0-1.5)	1.2(1.0-1.4)	1.6(1.4–1.8)	**< 0.001**
PAd (mm)	32(29.0-36.7)	32(29–37)	32(30.5–36.0)	0.408
RV wall thickness (mm)	5.3(4.5–6.2)	5.2(4.5–6.2)	5.4(4.6–6.4)	0.134
RVOT-Prox (mm)	36(32–40)	35(31–39)	38.5(36.0–42.0)	**< 0.001**
TR severity, n (%)				**< 0.001**
Non or Trace TR	33 (16.7)	31 (19.4)	2 (5.4)	
Mild TR	86 (43.6)	77 (48.1)	9 (24.3)	
Moderate TR	61 (30.9)	42 (26.2)	19 (51.4)	
Severe TR	17 (8.6)	10 (6.3)	7 (18.9)	
RVOT AcT (ms)	70.9 ± 17.9	73.6 ± 18.4	62.0 ± 12.0	**< 0.001**
TRV (cm/s)	425.8 ± 60.7	424.6 ± 59.1	431.5 ± 67.5	0.518
TRPG (mmHg)	73.8 ± 20.4	73.3 ± 20.5	76.1 ± 20.4	0.44
sPAP_Echo_ (mmHg)	79.8 ± 21.6	78.5 ± 21.7	85.1 ± 21.1	0.103
PREDV (cm/s)	292.7 ± 48.4	290.6 ± 45.7	298.7 ± 55.7	0.417
PREDG (mmHg)	35.0 ± 12.1	34.4 ± 11.3	35.1 ± 12.4	0.366
mPAP_Echo_ (mmHg)	40(33.0-48.7)	38(32.0-46.5)	45(38–52)	0.065
TVI_RVOT_ (cm)	10.3(7.8–12.6)	11.1(8.5–13.1)	7.7(5.5–9.2)	**< 0.001**
mRAP_Echo_ (mmHg)	3(3–8)	3(3–8)	8(3–15)	**< 0.001**
TAPSE (mm)	16(14.0-18.2)	16.4(14.7–19.0)	13.0(11.9–15.5)	**< 0.001**
S’ (cm/s)	9.6 ± 2.3	9.9 ± 2.3	9.5 ± 2.2	**0.001**
RV FAC (%)	29.9 ± 8.2	30.7 ± 8.4	26.8 ± 6.0	**0.014**
TAPSE/sPAP	0.19(0.15–0.26)	0.21(0.16–0.27)	0.15(0.12–0.19)	**< 0.001**
Pericardial effusion, n (%)	42(21.3)	26(16.2)	16(43.2)	**< 0.001**

PAP, pulmonary artery pressure; RAP, right atrial pressure; PAWP, pulmonary artery wedge pressure; PVR, pulmonary vascular resistance; CI, cardiac index; SvO_2_, mixed venous oxygen saturation; LVEF, left ventricular ejection fraction; LA, left atrial; LVIDd, left ventricular end-diastolic diameter; RA, right atrial; RV, right ventricular; RVEDA, right ventricular end-diastolic area; RVESA, right ventricular end-systolic area; LV, left ventricular; PAd, pulmonary artery diameter; RVOT-Prox, right ventricular outflow tract proximal diameter; TR, tricuspid regurgitation; RVOT Act, RVOT acceleration time; TRV, tricuspid regurgitation velocity; TRPG, tricuspid regurgitation pressure gradient; sPAP_Echo_, echocardiographic determination of systolic PAP; PREDV, The early diastolic pulmonary regurgitation velocity; PREDG, early diastolic pulmonary regurgitation pressure gradient; mPAP_Echo_, echocardiographic determination of mean PAP; TVI_RVOT_, velocity time integral of the right ventricular outflow tract; mRAP_Echo_, echocardiographic determination of mean RAP; TAPSE, tricuspid annular plane systolic excursion; S’, systolic annular tissue velocity of the lateral tricuspid annulus; RVFAC, right ventricular fractional area change;

### Risk score construction and internal validation

Binary variables were used to model the probability of high risk of pcPH by logistic regression. The echocardiographic parameters in Table 2 were initially considered as possible predictors and included in the model. The univariable and final multivariable logistic regression model retrieved through this approach was reported in Table 3. The final included predictors and thresholds were: the presence of pericardial effusion, RA area > 27 cm^2^, RVOT-Prox > 36 mm, TVI_RVOT_ < 10.1 cm, S’ < 7.3 cm/s.

**Table 3:** Univariable and multivariable logistic regression model for high risk of pre-capillary PH

Variable	Univariate analysis			multivariate analysis		
	OR (95%CI)	*P* value		OR (95%CI)	Wald	*P* value
Pericardial effusion	3.75 (1.74,8.11)	0.0008		2.403 (0.706, 8,183)	1.9672	0.1607
LVIDd (mm)	7.79 (3.31,18.31)	0.0011				
RA area (cm^2^)	3.59 (1.67,7.74)	< 0.0001		3.357 (1.042, 10.817)	4.1151	0.0425
RV basal diameter (mm)	9.60 (4.13,22.29)	0.0001				
RV/LV basal diameter ratio	4.60 (2.11,10.00)	< 0.0001				
RVOT-Prox (mm)	4.07 (1.75,9.45)	0.0011		3.517 (0.964, 12.836)	3.6264	0.0569
TVI_RVOT_ (cm)	8.92 (3.25,24.45)	< 0.0001		5.081 (1.325, 19.482)	5.6206	0.0178
mRAP_Echo_ (mmHg)	4.53 (1.98,10.36)	0.0009				
TRV (cm/s)	2.18 (1.04,4.56)	0.0382				
TAPSE (mm)	4.93 (2.31,10.53)	< 0.0001				
S’ (cm/s)	5.95 (2.47,14.33)	< 0.0001		6.128 (1.843, 20.37)	8.7494	0.0031
RV FAC (%)	4.00 (1.47,10.92)	0.0068				
TR severity	3.2 (1.53, 6.75)	< 0.0001				

LVIDd, left ventricular end-diastolic diameter; RA, right atrial; RV, right ventricular; LV, left ventricular; RVOT-Prox, right ventricular outflow tract proximal diameter; TVI_RVOT_, velocity time integral of the right ventricular outflow tract; mRAP_Echo_, echocardiographic determination of mean RAP; TRV, tricuspid regurgitation velocity; TAPSE, tricuspid annular plane systolic excursion; S’, systolic annular tissue velocity of the lateral tricuspid annulus; RVFAC, right ventricular fractional area change; TR, tricuspid regurgitation

**Fig. 3:**
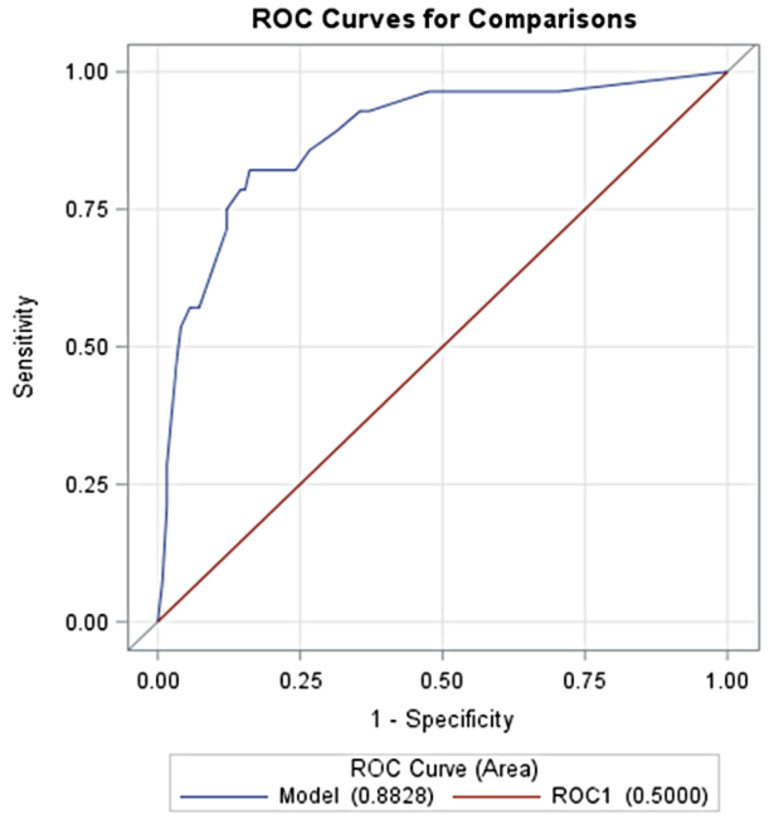
Receiver operation characteristic curves of the risk score model

The predictive performance of the overall model was assessed by the calculation of the AUC at ROC analysis, which was equal to 0.882 (95% CI: 0.809–0.956, *p* < 0.0001) (Fig. 3).

In order to simplify the use of the model in clinical practice, integer point scores were assigned to each predictor, which were derived from the β-coefficients in the final model (Table 4). Overall, there were 11 possible points in the risk score, with scores ranging from 0 to 10 in the training cohort. The estimate risk of high risk of pcPH ranged from 25.1% for those with a score of 0 to 94.6% for those with a score of 10 (Table 5).

**Table 4:** Score point assignment according to multivariable regression coefficients

Variable	β-coeffificient	Point
Pericardial effusion		
**Yes**	0.4384	1
**No**		0
RA area (cm^2^)		
**> 27**	0.6055	2
**≤ 27**		0
RVOT-Prox (mm)		
**> 36**	0.6289	2
**≤ 36**		0
TVI_RVOT_ (cm)		
**< 10.1**	0.8128	2
**≥ 10.1**		0
S’ (cm/s)		
**< 7.3**	0.9064	3
**≥ 7.3**		0

RA, right atrial; RVOT-Prox, right ventricular outflow tract proximal diameter; TVI_RVOT_, velocity time integral of the right ventricular outflow tract; S’, systolic annular tissue velocity of the lateral tricuspid annulus;

**Table 5:** The estimate risk of high risk of pre-capillary PH

Point	Estimate risk
0	0.251
1	0.342
2	0.446
3	0.556
4	0.660
5	0.750
6	0.823
7	0.878
8	0.918
9	0.946
10	0.964

The internal validity of the model was checked by bootstrapping. The model was confirmed in the ROC analysis with an AUC of 0.884. A total of 77 patients aged 57 ± 13 years were enrolled in the external validation cohort, consisting of patients with idiopathic pulmonary hypertension (*n* = 5) and CTEPH (*n* = 72). The AUC was found to be 0.852 with a sensitivity and specificity of 71% and 76%, respectively.

## Discussion

In the present study, we developed and internally validated an echocardiographic risk score model to predict the high risk of pcPH. We conducted a 10-point score to predict the estimate risk of high risk of pcPH. The echocardiographic scoring model comprehensively evaluated the status of pcPH patients from the aspects of RV morphology, blood flow and functional status in pcPH. We believe the echocardiographic risk score model would provide additional information for predict the prognosis and follow-up of pcPH.

For the initial risk stratification of newly diagnosed patients, what clinicians are hoping for is more indicators to participate in risk stratification and evaluate patients more comprehensively. As a convenient and noninvasive method, echocardiography can assess the condition of the cardiac from several aspects, including cardiac structure, function, and hemodynamic changes. However, due to the characteristics of ultrasonic technology, single echocardiographic indicator has their own limitations and is insufficient for providing comprehensive information. RA area and pericardial effusion were included in the 2015 and 2022 ESC/ERS Guidelines as imaging parameters associated with increased and decreased risk of adverse events [1, 2]. TAPSE/sPAP was added to the 2022 ESC/ERS Guidelines as a new echocardiographic indicator [1]. However, there is no conclusion in the previous studies and clinical practice on which conventional echocardiographic indicators are more valuable in the risk stratification of pcPH. A total of 5 indicators of our study were finally entered into the scoring model. The RA area and pericardial effusion are consistent with the guidelines. Our results showed that the cut-off value of RA area in high-risk pcPH was 27 cm^2^, which was similar to the value of 26 cm^2^ in the 2015 and 2022 guidelines. As a simple and easily obtained morphological index, RA area indirectly reflects the increase of RV filling pressure and the decrease of RV function. In a three-dimensional echocardiographic study by Julia et al., RA dilation is showed to be linked to an adverse clinical outcome [11]. Pericardial effusion is a sensitive index that can be detected by echocardiography and it is also an indirect sign of RV failure, which often indicates decompensation of RV function in PH patients. In a large group of pcPH study, pericardial effusion was one of the strongest predictors of mortality [12].

In addition to the two echocardiographic indexes included in the guideline, RVOT-Prox, TVI_RVOT_ and S’ are also included in our model. RV function is a key determinant of outcome in PH. Evaluation of RV function is crucial in clinical. Multiple parameters, including TAPSE, FAC, S’ as well as 3DE EF and longitudinal strain and strain rate have been demonstrated the clinical and prognostic value in assessment of RV systolic function [13–15]. TAPSE and S’ represent RV longitudinal function. TAPSE and S’ are easily obtained and convenient for routine application compared to the strain and strain rate we have studied before. They had been shown to correlate well with parameters estimating RV global systolic function. In our study, TAPSE was not included in the final multivariable logistic regression model. This may be related to the advantages of S’ itself. The tissue Doppler is relatively independent of image quality, and the peek speed of the spectrum is easy to identify. We speculate that these may result in S’ being more reproducible than TAPSE. Our previous research supported this hypothesis [16]. It is important to note that the cursor aligned along the direction of the tricuspid lateral annulus. RV FAC provides an estimate of global RV systolic function. However, the measurement of RV FAC has shown to be less reproducible as compared with TAPSE and S’ [8]. The cut-off value of S’ in high risk pcPH was 7.3 cm/s, which was significantly lower than the value published by the ASE for reduced right ventricular systolic function. This suggested that high risk pcPH had significantly worse RV systolic than low-intermediate risk pcPH.

TVI_RVOT_ by pulsed wave Doppler is also a convenient index for routine measurement and represents transpulmonary blood flow. The pulmonary vascular disease in PAH leads to RV dysfunction with reduced pulmonary and systemic blood flow. The occlusion of pulmonary arteries in CTEPH leads to increased PVR and progressive right heart failure, as well as reduced pulmonary blood flow. Therefore, severe pcPH patients with higher PVR have lower pulmonary blood flow. This may be the pathophysiological mechanism by which TVI_ROVT_ reflects disease severity and can reflect the severity of the disease in pcPH.

Our study showed that RVOT-Prox had significant weight in both univariable and final multivariable analyses and eventually entered the model, which was an unexpected new finding and also aroused our interest of this index. RV dimension evaluated by echocardiography have been analyzed in PAH studies, all proving their prognostic relevance [17, 18]. Anatomically, RVOT is part of the right ventricle. The 2010 ASE guide introduced the method of measuring the diameter of ROVT [9]. But compared with the inflow and trabecular regions mentioned in previous studies, few studies have paid attention to the diameter of RVOT. According to the results of our study, enlargement of ROVT may be an important structural change in right ventricular remodeling and reflects abnormal right ventricular function. The underlying mechanism could be as follows. Direct exposure of the RV outflow region to elevated pressure and resistance from the pulmonary vascular bed may lead to RVOT remodeling. Previous three-dimensional echocardiography study has showed that the increased afterload in PAH may be the cause for remodeling of the RVOT into a more circular shape [19]. We also observed enlargement of RVOT and marked hypertrophy of supraventricular ridge in PH patients. RVOT proximal diameter is relatively easy to measure and reproducible from basal short-axis view. If this is really a valuable indicator, it could be very convenient for routine use.

In addition to the five indicators obtained in our model, other indicators such as RV diameter, mRAP_Echo_, RV/LV, LVIDd, TAPSE, RV FAC and TR severity can all reflect the severity of the disease and also deserve our attention. These echocardiographic indicators differed significantly between high-risk and low-intermediate risk group and were also significant in the univariate analysis. Increased RV afterload results in RV enlargement. Reduced RV function further reduces transpulmonary blood flow affecting LV filling and corresponding with a significantly reduced LV diastolic volume in high-risk pcPH. Therefore, LV dimension can also indirectly reflect the severity of the disease in pcPH patients. RAP is considered as an important parameter to predict the prognosis of pulmonary hypertension. Patients with RAP > 14 mmHg were considered high risk in PAH [1, 2]. Echocardiographic estimation of mRAP is simpler and non-invasive compared with RHC. Functional TR is often secondary to RV enlargement and tricuspid ring enlargement. It has also been reported that TR severity correlated with PAH severity [20].

PAP is often one of the most concerned indicators for doctors and patients during echocardiographic evaluation. Our results showed that neither RHC nor echocardiographic measurement of pulmonary artery pressure can truly reflect the patient’s condition and predict risk stratification. Other studies have had similar results. In contrast to common belief, the estimated PAP is usually not prognostic and not relevant for therapeutic decision making [21, 22]. The estimated sPAP may be affected through the coupling mechanism between RV contractility and its load. During the initial phase of PH, RV coupling could be maintained by enhanced RV contractility. However, as PH progresses and RV uncoupling occurs, cardiac output would decrease, along with increase RAP, so the estimated PAP would be underestimated and not be a sign of disease improvement at this time. Our previous study also found this phenomenon [23]. TAPSE/sPAP ratio is a composite indicator that combines pulmonary pressure and RV function and is indeed better than a single index. It is a new echocardiographic indicator for risk stratification proposed in the 2022 guideline. TAPSE/sPAP has been proposed to be a non-invasive measurement of RV-arterial coupling [24, 25]. However, it may be limited when accurate sPAP and TAPSE cannot be obtained with non or trace TR or early post PEA patients. In addition, TAPSE/sPAP is a compound indicator, which will have too much weight in the multivariate analysis. Further study is needed on its prognostic value in patients with pcPH.

### Limitations

There are several limitations to the present study. First, our study subjects were pcPH, and the risk stratification of the subjects was according to the stratification in PAH. Patients with CTEPH formed the largest subgroup in this study. There is as yet no established risk stratification strategy in CTEPH. We identified high risk subjects according to a simplified risk stratification derived from the 2015 ESC/ERS model, which was validated in real-life conditions for PAH and CTEPH. However, the echocardiographic scoring model of this study does not apply to PH associated with left heart disease and may not apply to PH associated with lung diseases and congenital heart disease. Second, the sample size of this study and the number of events was relatively small. Further prospective studies involving larger patient population are required in the future. Finally, only conventional indexes were analyzed in this study, and the three-dimensional and speckle tracking indexes were not included. At present, the three - dimensional and speckle tracking index are not convenient for clinical application and follow-up. However, for some PH patients in our center, 3DRVEF, RVGLS, etc., will also be referred to as evaluation indicators.

## Conclusions

This study developed and validated a risk score based on five conventional echocardiographic parameters (pericardial effusion, RA area, RVOT-Prox, TVI_RVOT_ and S’) to predict high risk pcPH. Our scoring model comprehensively evaluated the RV status of pcPH in terms of RV morphology, RV function, and pulmonary blood flow. The multiparameter scoring index may allow better stratification of the newly diagnosed patient in clinical practice. It may also provide additional imaging information at follow-up for the basic four-strata model proposed in the 2022 ESC/ERS Guidelines.

## Data Availability

No datasets were generated or analysed during the current study.
